# Clinical evaluation of the effect of acentric double pedicle graft with and without the use of platelet-rich fibrin (PRF) on root coverage outcomes in class I and II Miller root recessions: A randomized clinical trial

**DOI:** 10.15171/japid.2018.010

**Published:** 2018-12-25

**Authors:** Nader Abolfazli, Masoumeh Faramarzi, Fariba Salehsaber, Aysan Shahmorad, Hadi Kokabi, Sohrab Amini

**Affiliations:** ^1^Department of Periodontics, Faculty of Dentistry, Dental and Periodontal Research Center, Tabriz University of Medical Sciences, Tabriz, Iran; ^2^Department of Prosthodontics, Faculty of Dentistry, Dental and Periodontal Research Center, Tabriz University of Medical Sciences, Tabriz, Iran; ^3^Faculty of Dentistry, Dental and Periodontal Research Center, Tabriz University of Medical Sciences, Tabriz, Iran

**Keywords:** Acentric double pedicle graft, Platelet rich fibrin, Miller's gingival recession

## Abstract

**Background:**

Acentric double pedicle graft is an alternative to double pedicle graft, which can improve clinical outcomes by removing tension in sutures. This study examined the effect of using platelet-rich fibrin (PRF) on the success rate of acentric double pedicle graft in treating patients with Miller Class I and II recessions.

**Methods:**

A total of 16 Miller Class I and II lesions were studied in 8 patients. The samples were divided into two groups in terms of PRF use: with PRF and without PRF. Indices, including recession depth, width of keratinized gingiva and pocket depth, were measured with a standard Michigan O probe with Williams marking. Six months later, Kolmogorov-Smirnov test and Wilcoxon nonparametric test were applied with SPSS17 to analyze data.

**Results:**

The recession depth, width of keratinized gingiva, and increased root coverage exhibited a significant difference between the two groups after surgery, but no significant difference was found in pocket depths.

**Conclusion:**

Applying PRF with acentric double pedicle graft reduced the recession depth, increased the width of keratinized gingiva and enhanced the extent of root coverage when compared with the situation where PRF was not used. Therefore, this study supports the use of PRF with acentric double pedicle graft in root coverage treatments.

## Introduction

Gingival recession is defined as the displacement of the gingival margin apical to the cementoenamel junction, exposing the root surfaces.^1,2^ Its prevalence among the adult population is between 20% and 100%,^3,4^ which occurs in almost all populations.^1^ The etiology of gingival recession is multifactorial^4^ and generally it is limited to one root surface, usually involving the buccal surface.^1^ Gingival recession might involve single or multiple teeth and it is almost associated with anatomical conditions of the soft tissue such as a defect in keratinized mucosa, chronic trauma, periodontal diseases, and areas susceptible to biofilm accumulation, including crowding or edges of defective restorations.^3^ Gingival recession can cause aesthetic problems, sensitization of teeth, exposed root surfaces, and thus higher incidence of root caries and cervical abrasions, as well as anatomical changes in the area, leading to health problems and even progression of periodontal diseases.^5^ Different surgical procedures have been introduced for treating gingival recession. In general, periodontal plastic surgery procedures for treating gingival recession can be divided into four general categories:

1. Free gingival grafts (FGG)

2. Pedicle graft

3. Subepithelial connective tissue graft combined with pedicle graft

4. Guided tissue regeneration (GTR)^6^

The coronally advanced flap (CAF) is an advanced pedicle graft with connective tissue graft considered as the gold standard for root coverage.^7^ The use of this flap has some disadvantages, however. For example, when the depth of the vestibule is insufficient, this flap cannot be used; the flap itself can also reduce the depth of the vestibule. On the other hand, use of CTG causes more pain and discomfort for the patient.^8,9^

The double pedicle graft technique, introduced by Rose and Cohen in 1968, is a type of rotational pedicle graft. The advantages of this method include less trauma, excellent color coordination, and no impact of the vestibule depth on the results. Unfortunately, this method has also some disadvantages; the use of this technique is only possible when the interdental papilla has a significant mesiodistal thickness and width. Furthermore, it cannot be used in multiple defects. Another disadvantage of this method is that the connective tissue is not used. In addition, since the mesial and distal papillae are stitched at the midline of the exposed root surface with the highest tension, soft tissue dehiscence might compromise the results.^10^

Because of the disadvantages of this method, Harris tried to use this method along with connective tissue graft. According to his various studies in 1992, 1994 and 2005, connective tissue grafts combined with double pedicle grafts yielded results similar to the gold standard outcomes. However, one of its disadvantages was the need for a second surgical site for acquisition of connective tissue.^11-14^

Due to the disadvantages of the DPG method, the acentric double pedicle graft technique was introduced by Abolfazli et al. In the DPG method, both papillae are cut horizontally and the two edges of the flap reach each other in the mid-root region, causing tension in sutures given their placement on the height of contour of the teeth. However, in acentric double pedicle graft technique, one of the mesial and distal papillae is cut obliquely; then another papilla is cut horizontally as with in DPG. Finally, the two edges of the flap reach each other and are sutured in the mesial or distal region. This way, this method removes tension at the site of sutures and prevents scarring and relapse of the treatment results.^15^

Nevertheless, use of connective tissue graft has also some disadvantages, including the need for a second surgical site for taking grafts, postoperative pain at the graft site, and the need for extensive graft in multiple gingival recessions.^16,17^ In efforts to find an alternative to connective tissue graft, various grafts and biomaterials have been suggested, including blood derivatives such as Platelet Rich Plasma (PRP) and Platelet Rich Fibrin (PRF) as well as AlloDerm grafts.^18^ The common feature of these alternatives is the avoidance of second surgery for taking a connective tissue, reducing the patient's discomfort after surgery, adequate availability, and saving time.^19^

Studies on platelets often deal with their role in homeostasis. Notably, in addition to homeostasis, they have another physiological function which has recently been studied.^20^ Platelets are various growth factor carriers, which play an essential role in the repair and regeneration of soft tissues.^21^ Several studies have shown that growth factors in plasma stimulate the process of repairing and regenerating soft and hard tissues and reducing the inflammation and subsequent pain. Among these growth factors and cytokines, platelet-derived growth factor (PDGF), vascular endothelial growth factor (VEGF), transforming growth factor beta (TGF-β), and platelet-derived epidermal growth factor (PD-EGF) have been noted.^20^

Platelet-rich plasma (PRP) and platelet-rich fibrin (PRF) are autologous platelet-rich plasma obtained from patient's own blood. Recent studies focusing on the development of alternative therapies in periodontal plastic surgeries suggest that the process of using PRF is easier, cheaper, and more biocompatible than PRP, and releases more growth factors over a longer period of time. Overall, it yields better outcomes compared to PRP.^22^

In a study by Jankovic,^19^ fifteen patients with bilateral Miller class I and II gingival recession defects in the anterior and premolar regions were studied. All the patients underwent bilateral gingival recessions surgery. The lesions on one side of the patients’ jaw were treated with CAF + PRF (PRF), while the other side was treated with CAF + CTG (control group). The results of this study demonstrated the success of both CTG and PRF methods, in combination with CAF. The advantages of PRF in this study were related to dispensing with donor site surgical procedure, improved wound tissue repair in the first two weeks following surgery, and significant reduction in patient's discomfort during wound healing.

In a systematic review and meta-analysis by Moraschini et al,^11^ the use of PRF membrane was evaluated in treating patients with Miller Class I and II gingival recession defects. Six RCT studies and one prospective study with a minimum six-month follow-up were included in this study. The results suggested that the use of PRF membrane did not improve clinical outcomes (root coverage, attached gingival width and gingival attachment limit) compared to other treatment techniques in treating Miller Class I and II gingival recession.

Gupta et al^23^ examined the clinical effect of CAF alone and in combination with PRF in 30 Miller Class I and II gingival recessions. The parameters of pocket depth, recession depth, loss of clinical attachment level, keratinized tissue width and gingival tissue thickness were measured before the treatment as well as 3 and 6 months after it. Complete root coverage was obtained in 12 cases in the test group and 11 cases in the control group, with no significant difference. The researchers reported that the CAF combined with PRF had no additional advantages in treating Miller Class I and II recessions.

In a systematic review, Ogata et al^24^ compared the effects of using PRF membrane on the clinical outcomes of treating Miller Class I and II gingival recessions with other methods. This study included 7 RCT and prospective controlled studies with a minimum 6-month follow-up. The results of this study indicated that the use of PRF membranes did not improve the clinical outcomes of Miller Class I and II gingival recession treatment, including root coverage, gingival attachment level and attached gingival width, compared with other methods.

In a study by Oncu^25^ to assess the clinical impact of PRF combined with Modified CAF and its comparison using SCTG combined with MCAF in treating multiple and bilateral Miller Class I and II gingival recessions, 20 patients participated in a split-mouth randomized controlled trial. The gingival recession depth (RD), keratinized tissue width (KW), probing depth (PD), clinical attachment level (CAL), and gingival thickness (GT) were evaluated before surgery and 6 months after it. It was reported that treatment of gingival recession was successful in both groups and the PRF advantage enhanced patient comfort in the postoperative period. The researcher suggested that the use of PRF is an appropriate alternative to SCTG in the treatment of gingival recession.

Due to the disadvantages of using connective tissue graft on the root surface and the use of new surface-covering materials, there is an information gap on the use of these materials in the acentric double pedicle graft technique.

Furthermore, owing to the lack studies on the effect of PRF on the clinical outcomes of covering the exposed roots in the pedicle graft, we decided to evaluate the effect of acentric double pedicle graft on patients with Miller Class I and II gingival recessions. The specific objectives of this study included the evaluation and comparison of changes in the width of keratinized gingiva, recession depth and pocket depth after using acentric double pedicle graft with and without PRF.

## Methods

This study was a clinical trial and the samples were selected from patients referring to the Department of Periodontics in Tabriz Faculty of Dentistry. The sample size was determined according to Harris’s study and by considering the width of keratinized gingiva.^12^ By using two methods of 1,1 units with standard deviation of 1.4 and also considering the αu=0.05 and the 80% power, the sample size was calculated at15. In order to increase the validityof the study and the possibility of losing samples, the sample size was increased by 10% and 16 samples were included.

The study had a split-mouth design, in which 16 lesions of Miller Class I and II were studied in 8 patients in terms of predictable root coverage treatments in these types of recessions in the buccal/facial surface, with a depth of at least 2 mm, as well as in canine space, premolar or maxillary, and mandibular incisors. The existence of adequate vestibular depth was a prerequisite for surgery. Also, if the patient had a high frenal attachment, it was resolved.

This study was a single-blind study in which the surgeon was aware of the allocation but the patients were blinded to the side in which PRF was used. Also the randomization method was simple randomization and the samples were grouped by rolling of a die. The die was rolled for one of the patient’s involved teeth. Numbers 1,3 and 5 represented that the lesion of the considered tooth was going to be treated using PRF and numbers 2,4 and 6 represented that the other lesion gets treatment without PRF.

The inclusion criteria for the study included:

1. Vital teeth without a history of endodontic treatment

2. No history of any surgical intervention in the area during the past two years

3. Signing written consent by the patient prior to surgery after explaining all the stages of surgery to the patient

4. Being non-smoker^13^

On the other hand, the exclusion criteria included:

1. Systemic diseases affecting the periodontium

2. Bleeding on probing in the surgical areas

3. Root-surface restorations in the test or control teeth

4. A plaque index of ≥20

5. Pregnancy

6. History of corticosteroid therapy

7. A history of root coverage surgery on the test or control teeth

8. Need for prophylaxis due to systemic problems^13^

The surgical procedure was the same in both the case and control groups. Accordingly, eligible patients received 800 mg of ibuprofen before surgery.^13^ Vertical recession depth, keratinized tissue width and pocket depth were measured before surgery in the tooth medial area by a blinded calibrated examiner using the standard Williams Marking Michigan Probes. Local anesthesia was performed with 2% lidocaine hydrochloride solution with 1:100,000 epinephrine by infiltration technique. The root debridement was then performed using manual and ultrasonic instruments.

The preparation of PRF was performed using the method suggested by Choukroun et al.^26^ Accordingly, the patient's blood sample was drawn prior to surgery and placed in 10-mL test tubes without anticoagulants and immediately centrifuged at 3000 rpm for 10 minutes. During centrifugation, the thrombin in the blood converts fibrinogen to fibrin, which is placed in the middle of the tube. PRF resulting from centrifugation is produced in two forms: I) in gelatinous and amorphous form; II) as a stable fibrin membrane, which was used in this study.

In order to perform surgery, oblique incisions were made in the mesial and distal of the teeth with gingival recession, parallel to the CEJ of the adjacent teeth with a No.15 blade. Specifically, the primary incision was made more apically than the mesial or distal papilla parallel to the root of the adjacent tooth and extended to the CEJ. Then, contrary to the first incision which was parallel to the root of the adjacent tooth, a horizontal incision was made below another papilla at a distance of at least 0.5 mm from the marginal gingiva of the adjacent tooth. Vertical incisions were then made perpendicularly to the horizontal incisions such that the beginning of these incisions was right at the end of the horizontal incisions and was extended to the alveolar mucus. Then, the flap was returned as close as possible to the periosteum.^15^ The returning flap was placed to the extent that the mesial and distal parts of the flap had freedom of movement. The mesial and distal parts of the flap were placed on the gingival recession area where they remained attached without maintenance.

**Figure 1:**
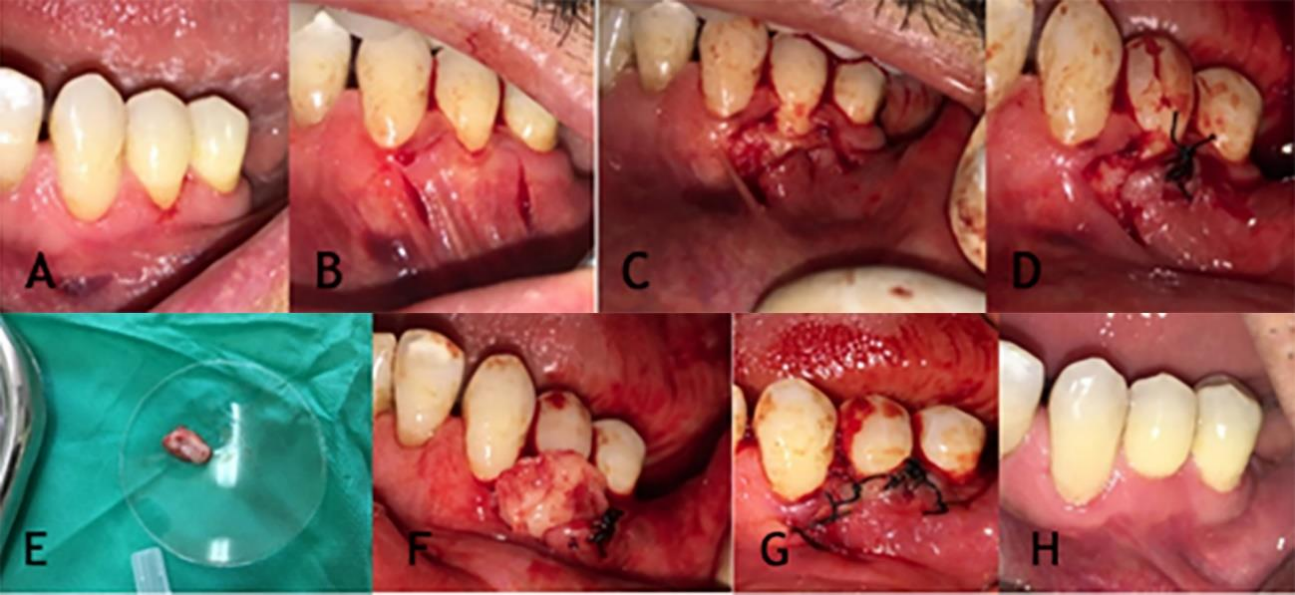
Steps of root coverage surgery using Acentric double pedicle graft with PRF.

**Figure 2:**
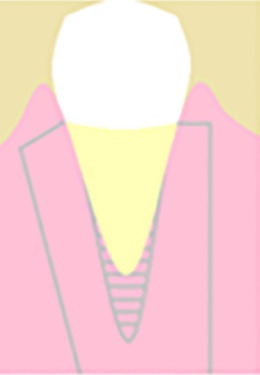
Schematic representation of the location and orientations of incisions.

Any existing tissue tags were removed, and after placing the PRF membrane on the gingival area, the membrane was sutured with catgut suture, after which the acentric flap was closed with silk sutures (0-5). No membranes were used in the control group (Figure 1 A to H & Figure 2). All parameters were measured at first & third month after surgery.

The results of the study were reported using descriptive statistics (means ± standard deviations and frequencies). When the data distribution was normal, paired t-test was used to compare the root coverage, width of keratinized gingiva, pocket depth and recession depth in the groups with and without PRF. On the other hand, when the data distribution was abnormal, Wilcoxon's test was employed. Furthermore, Kolmogorov-Smirnov test was utilized to evaluate the data normality and statistical analysis was performed using SPSS17. The significance level was considered at P<0.05.

An informed consent form was signed by each patient to be included in this research. The patients’personal information was kept confidential and if there was no more cooperation the patients were allowed to leave the study without any explanation. Also the study had no extra cost for the patients. The Ethics Committee of Tabriz Faculty of Dentistry approved the study protocol under the code IRCT20120805010501N5.

## Results

In this split-mouth clinical trial, 16 Miller Class I and II lesions were studied in 8 patients. The studied teeth in each patient and the clinical indices are fully reported in Table 1, including the RD, PD, and KW before gingival surgery in both PRF and non-PRF groups.

**Table 1:** Baseline demographic and clinical characteristics of the study group

**Patient**	**Sex**	**Age**	**Tooth number**	**RD (mm)**	**PD (mm)**	**KW(mm)**
1	M	26	21	2.5	1.5	0
1	M	26	25	4	3	0
2	F	41	4	3.5	1	0.5
2	F	41	12	2.5	2.5	1
3	F	32	5	3	2.5	1
3	F	32	12	2	1	2
4	M	37	21	4	2	2
4	M	37	27	3	0.5	1
5	F	29	7	3.5	2	1
5	F	29	10	2.5	1.5	2
6	F	35	28	5	2	2
6	F	35	20	3	2	1
7	M	41	20	2	1	1.5
7	M	41	28	3.5	2	2
8	M	27	28	2	1	0.5
8	M	27	20	3.5	2	0

According to the results of Kolmogorov-Smirnov test, the data distribution was not normal (P<0.05). Therefore, Wilcoxon's nonparametric test was used to compare the means of the indices before and after surgery and between the two PRF and non-PRF groups.

The comparative analysis of the measured indices is presented in Table 2. According to the results presented in this table, in the PRF group, there was a significant difference in recession depth (P=0.011), pocket depth (P=0.041) and width of keratinized gingiva (P=0.011) before and after surgery.

**Table 2:** Clinical measurements in patients before and after surgery

**Patient**	**Group**	**Teeth number**	**RD (mm)**		**PD (mm)**		**Root Coverage (%)**	**KW(mm)**	
			**Before**	**After**	**Before**	**After**		**Before**	**After**
**1**	with PRF	21	2.5	0	1.5	0.5	100	0	3
**1**	without PRF	25	4	2	3	2	50	0	2.5
**2**	with PRF	4	3.5	0.5	1	1	85	0.5	3.5
**2**	without PRF	12	2.5	1	2.5	1	60	1	4
**3**	with PRF	5	3	0	2.5	1	100	1	4
**3**	without PRF	12	2	1	1	1	50	2	4
**4**	with PRF	21	4	0.5	2	0.5	87.5	2	5
**4**	without PRF	27	3	0.5	0.5	0.5	83	1	2.5
**5**	with PRF	7	3.5	0	2	1	100	1	5
**5**	without PRF	10	2.5	1	1.5	1	60	2	4
**6**	with PRF	28	5	0.5	2	2	90	2	4.5
**6**	without PRF	20	3	0.5	2	0.5	83.3	1	4
**7**	with PRF	20	2	0	1	1	100	1.5	3.5
**7**	without PRF	28	3.5	0.5	2	1	853.7	2	3
**8**	with PRF	28	2	0	1	0.5	100	0.5	3
**8**	without PRF	20	3.5	0.5	2	0.5	83.3	0	1.5

In the non-PRF group, the results were similar and there were significant differences in the three indexes of recession depth (P=0.011), pocket depth (P=0.026) and width of keratinized gingiva (P=0.011) before and after the surgery.

In the PRF and non-PRF groups, the mean values of root coverage were 95.31% and 69.41%, respectively. This index was compared in both PRF and non-PRF groups by Wilcoxon test, which indicated a significant statistical difference between the mean value of root coverage in the two groups (P=0.012).

In order to compare the indices measured in both groups, there should be no significant difference between the measured values of each index before surgery so that the difference between the mean values of postoperative indexes obtained between the two groups could be attributed to the intervention. This comparison was also performed using the Wilcoxon test. This condition was established in all the three indices and the mean value of preoperative measurements between the two groups was not statistically significant.

(P[KW]=0.862) (P[PD]=0.730) (P[RD]=0.774).

Accordingly, Wilcoxon test was employed to compare the mean value of measures in the two groups, with the results presented in Table 3 & Table 4. The recession depth in the PRF group was 0.68 mm less than that in the non-PRF group with the difference being statistically significant (P=0.026). The pocket depths in both PRF and non-PRF groups were the same, with no statistically significant difference (P=0.999). The mean value of keratinized gingiva width in the PRF group was 0.75 mm greater than that in the non-PRF group, and this difference was statistically significant (P=0.048).

**Table 3:** Comparison of indices measured in both groups before and after gingival surgery

			**Min**	**Max**	**Mean±SD**	**Mean difference before and after**	**P-value**
**Group with PRF**	**RD**	Before	2	5	3.18±1.03	3	0.011
		After	0	0.5	0.18±0.25		
	**PD**	Before	1	2.5	1.62±0.58	0.68	0.041
		After	0.5	2	0.93±0.49		
	**KW**	Before	0	2	1.06±0.72	-2.78	0.011
		After	3	5	3.93±0.82		
	**Root Coverage**		85%	100%	95.31%	---	---
**Group without** **PRF**	**RD**	Before	2	4	2.93±0.62	2.06	0.011
		After	0	2	0.87±0.51		
	**PD**	Before	0.5	3	1.81±0.79	0.87	0.026
		After	0.5	2	0.93±0.49		
	**KW**	Before	0	2	1.12±0.83	-2.06	0.011
		After	1.5	4	3.18±0.96		
	**Root Coverage**		50%	85.7%	69.41%		---

**Table 4:** Comparison of measured indices in both PRF and non-PRF groups after gingival surgery

		**Mean±SD**	**Mean difference**	**P-value**
**RD**	with **PRF**	0.18±0.25	-0.68	0.026
	without **PRF**	0.87±0.51		
**PD**	with **PRF**	0.93±0.49	0	0.999
	without **PRF**	0.93±0.49		
**KW**	with **PRF**	3.93±0.82	0.75	0.048
	without **PRF**	3.18±0.96		

**Figure 3:**
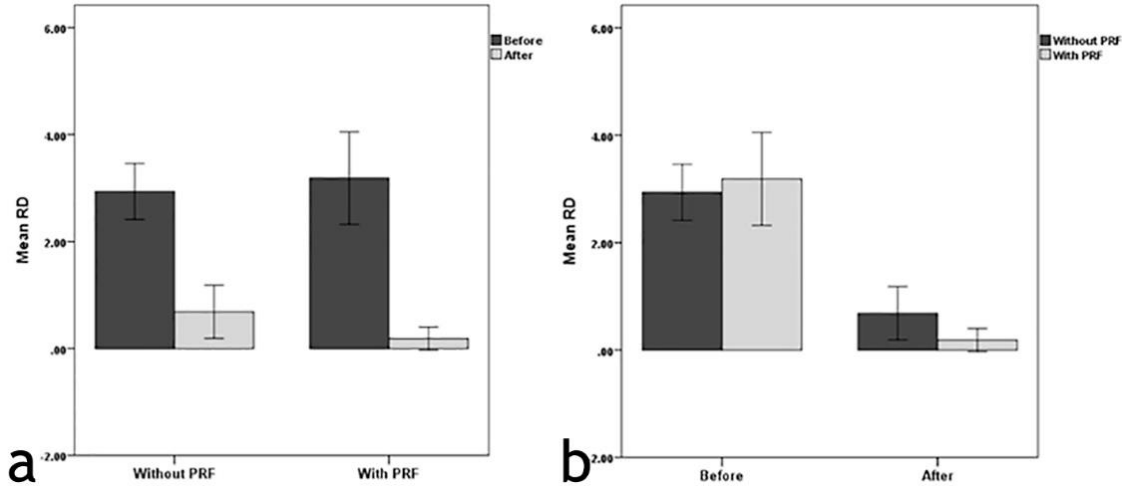
Comparison of gingival recession depth (RD) in groups with and without PRF, before and after surgery.

Figures 3a and 3b compare the mean magnitudes of RD in the two groups and the two time intervals.

**Figure 4:**
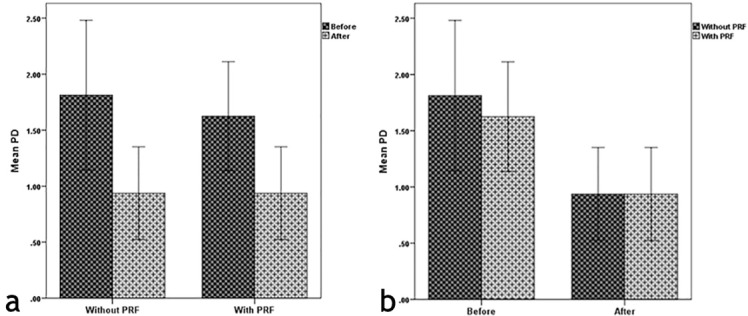
Comparison of probing depth (PD) in groups with and without PRF, before and after surgery.

Figures 4a and 4b compare the mean PD values between the two groups and two time intervals.

**Figure 5:**
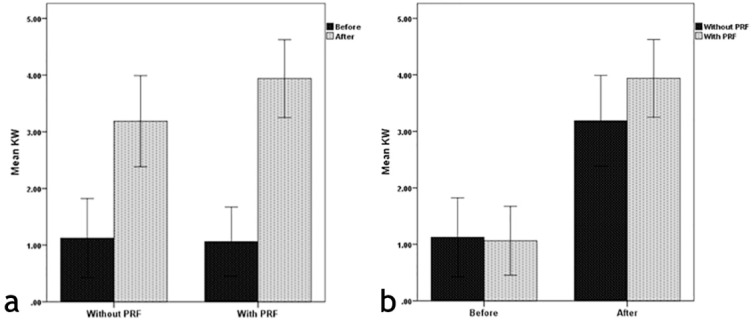
Comparison of keratinzed tissue width (KW) in groups with and without PRF, before and after surgery.

Figures 5a and 5b compare the mean KW values between the two groups and two time intervals.

**Figure 6:**
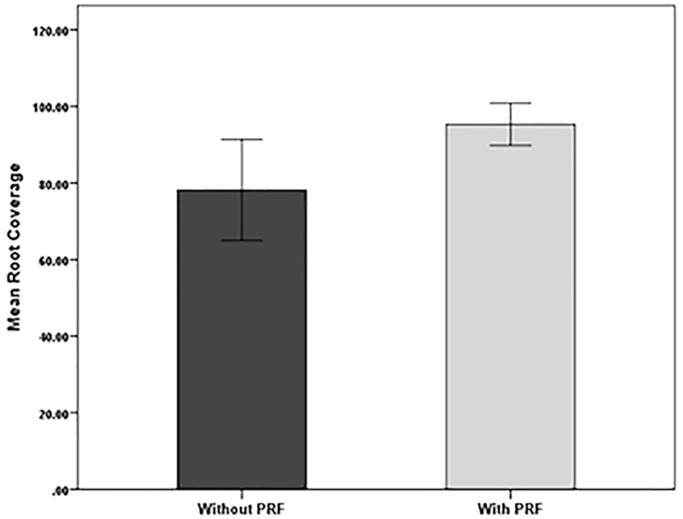
Comparison of mean root coverage in groups with and without PRF.

Figure 6 presents the mean values of root coverage between the two groups.

## Discussion

This study aimed to evaluate the clinical results of using acentric double pedicle graft with and without PRF in the coverage of exposed root surfaces with Miller Class I and II gingival recessions.

The results of this study clearly indicated that in both the PRF and non-PRF groups, all the three indices ‒ recession depth, pocket depth, and width of keratinized gingiva ‒ significantly changed after surgery. Specifically, in both groups the recession depth and pocket depth diminished while the width of keratinized gingiva increased (P<0.05), suggesting that intervention in these two groups was effective. On the other hand, a comparison between the two groups revealed that the decrease in the recession depth in the PRF group was significantly higher than that in the non-PRF group, which is statistically significant. Also, the width of keratinized gingiva in the PRF group increased significantly more than that in the group without PRF, which is also statistically significant (P<0.05). However, there was no significant difference between the two groups in terms of reduction in pocket depth (P<0.05).

Harris used the primary DPG technique to treat Miller Class I and II gingival recession lesions and reported 50% root coverage after 6 months,^12^ which was weaker than the results of applying ADPG alone in this study (69.41%). This difference can be attributed to the design of the incisions in the two methods and the location of sutures. In the primal DPG technique, two horizontal incisions and two vertical incisions are used, which produce interdental papillae of approximately the same size at the lesions of mesial and distal surfaces. Then, the intermediate mesial and distal papillae are displaced in the middle of the root and ultimately sutured in the middle region of the root.^12^ However, in the ADPG technique introduced by Abolfazli and Saber, the locationof suture is displaced to one side of the middle region of the root, which is, in fact, the height of contour, and the place of the greatest tension to separate mesial and distal interdental papillae, which probably has contributed to the success of the ADPG technique compared to DPG. Abolfazli and Saber reported success rates of 82% with this technique.^15^

Harris addressed the success rate of DPG + CTG to resolve DPG defects, where the root coverage percentage was 97.7%.^13^ The results of the present study also showed that the root coverage percentage was 95.31% with the use of the ADPG + PRF technique, which is very close to the results of Harris study. Since in the current technique, connective tissue is not removed from the palate, ADPG + PRF technique can be introduced as an alternative technique.

By comparing the results of CAF + CTG or CAF + PRF with ADPG + PRF, it can be concluded that both techniques result in similar success rates in root coverage. However, since the ADPG + PRF technique has no effect on the depth of the vestibule, this technique can be introduced as an alternative to the above-mentioned techniques. Note that failure to apply CTG does not cause pain and discomfort to the patient in the second place of surgery, and the use of PRF reduces inflammation and pain in the surgical area. On the other hand, by creating a fibrin matrix, it causes more angiogenesis and presence of immune factors, accelerating tissue regeneration in the surgical area.^25^

The results of this study revealed a significant increase in the width of keratinized gingiva in both the PRF and non-PRF groups compared to preoperative counterparts. Note that the increase in the width of the keratinized gingiva in the PRF group was significantly higher than that in the non-PRF group.

Studies by Jankovic, Moraschini, and Oncu suggested that the increase in the width of the keratinized gingiva in the CAF + CTG group was greater than that in the CAF + PRF group. Specifically, the growth in the mean width of keratinized gingiva was 1.6 mm in the CAF + CTG group.^11,19,25^ In this study, in the PRF group, the increase in the width of keratinized gingiva was 2.87 mm. The greater elevation of the width of keratinized gingiva in this study can be attributed to the results of ADPG technique, which itself increases the width of the keratinized gingiva. On the other hand, although an important feature of DPG is the increased width of keratinized gingiva, the results obtained in the study suggest a further increase in the width of keratinized gingiva in the PRF group. This can be attributed to better results in root coverage using PRF or because of the release of growth factors and cytokines continuing for 7 to 14 days after using PRF. This leads to greater tissue proliferation and increased width of keratinized gingiva.^27,28^ Based on the statistically insignificant difference in pocket depth reduction in both the PRF and non-PRF groups, it can be concluded that the use of PRF does not have any effect on pocket depth reduction. The results of this study suggested the positive effect of ADPG + PRF technique on the treatment of Miller class I and II gingival recessions. Considering the limited number of patients treated with this method and the relatively short period, it is suggested that the results of the study be examined using a larger sample size and for longer intervals. Some other limitations of this method can be summarized as follows:

1. It is highly sensitive and requires well-trained clinicians.

2. It is not applicable in multiple gingival recessions.

3. In this method, the width of keratinized gingiva at interdental papilla adjacent to the recession region should be 2‒3 mm larger than the area of gingival recession.

4. The study did not include a sufficient sample size.

5. The follow-up time was short in this research.

## Conclusion

At the beginning of the study, it was expected that using PRF would improve the clinical outcomes of the study.

As expected, the use of acentric double pedicle graft with PRF reduced the recession depth, increased the width of keratinized gingiva, and increased the coverage of exposed root surfaces compared to acentric double pedicle graft without PRF.

Despite the expectations, utilization of acentric double pedicle graft with PRF did not further reduce the pocket depth compared to acentric double pedicle graft without PRF.

This study supported the use of PRF with acentric double pedicle graft in root coverage treatments.

## Authors’ contributions

The study was designed by NA and SA. Data collection was performed by MF and SA; statistical analyses and interpretation of data were conducted by HK and SA. The manuscript was prepared by AS and SA and revised by NA and FS. Each author has read and approved the final manuscript for submission.

## Competing interests

The authors state that they have no competing interests with regards to the authorship and/or publication of this paper.

## Ethics approval

The Ethics Committee of Tabriz University of Medical Sciences approved the study protocol.
